# Impaired Systemic Tetrahydrobiopterin Bioavailability and Increased Oxidized Biopterins in Pediatric Falciparum Malaria: Association with Disease Severity

**DOI:** 10.1371/journal.ppat.1004655

**Published:** 2015-03-12

**Authors:** Matthew P. Rubach, Jackson Mukemba, Salvatore Florence, Bert K. Lopansri, Keith Hyland, Alicia D. Volkheimer, Tsin W. Yeo, Nicholas M. Anstey, J. Brice Weinberg, Esther D. Mwaikambo, Donald L. Granger

**Affiliations:** 1 Department of Medicine, Duke University and VA Medical Centers, Durham, North Carolina, United States of America; 2 Department of Pediatrics, Hubert Kairuki Memorial University, Dar es Salaam, United Republic of Tanzania; 3 Department of Medicine, Intermountain Healthcare, Salt Lake City, Utah, United States of America; 4 Department of Medicine, University of Utah School of Medicine and VA Medical Center, Salt Lake City, Utah, United States of America; 5 Neurochemistry Division, Medical Neurogenetics, Atlanta, Georgia, United States of America; 6 Global and Tropical Health Division, Menzies School for Health Research and Charles Darwin University, Darwin, Australia; 7 Division of Medicine, Royal Darwin Hospital, Darwin, Northern Territory, Australia; 8 Department of Medicine, Lee Kong Chian School of Medicine, Nanyang Technological University, Singapore; Albert Einstein College of Medicine, UNITED STATES

## Abstract

Decreased bioavailability of nitric oxide (NO) is a major contributor to the pathophysiology of severe falciparum malaria. Tetrahydrobiopterin (BH_4_) is an enzyme cofactor required for NO synthesis from L-arginine. We hypothesized that systemic levels of BH4 would be decreased in children with cerebral malaria, contributing to low NO bioavailability. In an observational study in Tanzania, we measured urine levels of biopterin in its various redox states (fully reduced [BH_4_] and the oxidized metabolites, dihydrobiopterin [BH_2_] and biopterin [B_0_]) in children with uncomplicated malaria (UM, n = 55), cerebral malaria (CM, n = 45), non-malaria central nervous system conditions (NMC, n = 48), and in 111 healthy controls (HC). Median urine BH4 concentration in CM (1.10 [IQR:0.55–2.18] μmol/mmol creatinine) was significantly lower compared to each of the other three groups — UM (2.10 [IQR:1.32–3.14];p<0.001), NMC (1.52 [IQR:1.01–2.71];p = 0.002), and HC (1.60 [IQR:1.15–2.23];p = 0.005). Oxidized biopterins were increased, and the BH4:BH2 ratio markedly decreased in CM. In a multivariate logistic regression model, each Log10-unit decrease in urine BH4 was independently associated with a 3.85-fold (95% CI:1.89–7.61) increase in odds of CM (p<0.001). Low systemic BH4 levels and increased oxidized biopterins contribute to the low NO bioavailability observed in CM. Adjunctive therapy to regenerate BH4 may have a role in improving NO bioavailability and microvascular perfusion in severe falciparum malaria.

## Author Summary

Vascular nitric oxide (NO) bioavailability is decreased in severe falciparum malaria and associated with microvascular dysfunction, increased activation of the cells lining blood vessels (endothelial cells) and increased parasite biomass. Tetrahydrobiopterin (BH_4_) is an essential cofactor for nitric oxide synthase (NOS) enzymatic conversion of L-arginine to NO and L-citrulline. But when BH_4_ is low, NOS is “uncoupled” and produces superoxide instead of NO. In oxidative conditions, BH_4_ is oxidized to dihydrobiopterin (BH_2_) and biopterin (B_0_). BH_2_ competes with remaining BH_4_ at its NOS binding site, further decreasing NOS-catalyzed NO production. We measured BH_4_, BH_2_ and B_0_ in the urine of children with coma due to falciparum malaria (cerebral malaria), uncomplicated falciparum malaria, children with non-malaria central nervous system conditions and healthy controls. Urine BH_4_ was significantly decreased and BH_2_ significantly increased in cerebral malaria compared to uncomplicated malaria, non-malaria central nervous conditions and healthy controls, suggesting increased oxidative stress and insufficient recycling of BH_2_ back to BH_4_. Urine BH_4_ concentration was independently associated with increased risk of cerebral malaria. Given that safe therapies for regenerating BH_4_ have been studied in chronic vascular disease, this finding of low BH_4_ in pediatric cerebral malaria offers a new area of investigation for adjunctive therapies aimed at improving NO bioavailability and, consequently, clinical outcomes in severe falciparum malaria.

## Introduction

Falciparum malaria causes over 600,000 deaths worldwide each year, with approximately 560,000 fatal cases annually among children in sub-Saharan Africa [1]. Coma in malaria, cerebral malaria (CM), portends a grave outcome among children infected with *Plasmodium falciparum* and, despite advances in anti-parasitic drug therapies, still has a 10–20% case fatality rate [2–4]. However, the pathogenesis of CM remains poorly understood [5]. CM pathogenesis studies to date show endothelial dysfunction [6], endothelial activation [7–10] and cytoadherence of parasitized red blood cell (pRBC) to endothelial cells in post-capillary venules, resulting in red blood cell sequestration [11–13], microvascular congestion and impaired blood flow to tissues [14–16]. Metabolic derangements and cytotoxic mechanisms contributing to the pathogenesis of CM have also been proposed [5,17–19].

Low nitric oxide (NO) is a key cause and contributor to the microvascular pathophysiology observed in severe malaria [20]. A variety of causes for low bioavailability in malaria have been identified within multiple steps of the NO production pathway [21–23], from low levels of NO synthase (NOS) substrate, arginine [24], to elevated levels of endogenous NOS inhibitors [25,26]. All NOS isoforms require the obligate cofactor tetrahydrobiopterin (BH_4_) to enzymatically generate NO from L-arginine. The role of this NOS cofactor as a potential contributor to low NO bioavailability in malaria is unknown. In vascular diseases, low BH_4_ and increased concentrations of its oxidized metabolite, dihydrobiopterin (BH_2_), are not only associated with impaired NO synthesis, but also linked to generation of reactive oxygen species within the endothelium [27].

In addition to its role in NO synthesis and endothelial function, BH_4_ is also an essential cofactor for monooxygenase enzymes required for phenylalanine metabolism (phenylalanine hydroxylase [PAH]) as well as biogenic amine neurotransmitter synthesis of catecholamines (tyrosine hydroxylase) and serotonin (tryptophan hydroxylase) [28,29]. (The reader is directed to references 28 and 29 for reviews of BH_4_ metabolism, which include diagrams of BH_4_ synthetic and salvage pathways.) We have previously reported elevated plasma phenylalanine in children with CM [18]. This finding is likely attributable to impaired activity of hepatic PAH, which regulates plasma phenylalanine levels within a narrow range by controlling the rate of conversion of phenylalanine to tyrosine [30]. We hypothesized that hyperphenylalaninemia is an indicator of systemic BH_4_ deficiency and that BH_4_ deficiency would also contribute to impaired NO bioavailability observed in malaria.

To address this hypothesis we quantified BH_4_ and its oxidized metabolites in urine as a measure of systemic biopterin availability [28,31–33] in children presenting with CM. Quantifying biopterins in urine requires specific collection methods. With ordinary urine collection and storage, BH_4_ spontaneously oxidizes to its metabolites, BH_2_ and, to a lesser extent, fully oxidized biopterin (B_0_) [34,35]. By collecting urine directly into an anti-oxidant/chelator cocktail in the dark followed by immediate freezing and storage at -80°C until analysis, we overcame this potential artifact of *ex vivo* spontaneous oxidation. This established collection method for biopterin analysis enabled quantification of BH_4_ in its reduced *in vivo* state [35]. In this report, we provide quantitative data on urinary excretion of the active NOS cofactor BH_4_, as well as BH_2_ and B_0_, in children at the time of presentation with CM. We found decreased levels of BH_4_ and increased levels of oxidized biopterins in children with CM, indicating a marked reduction in BH_4_ bioavailability in severe malaria. Based on these findings, we propose a pathogenic mechanism wherein low systemic BH_4_ contributes to low NO bioavailability and endothelial dysfunction in severe malaria.

## Results

### Clinical variables describing the clinical groups

**Fig 1:**
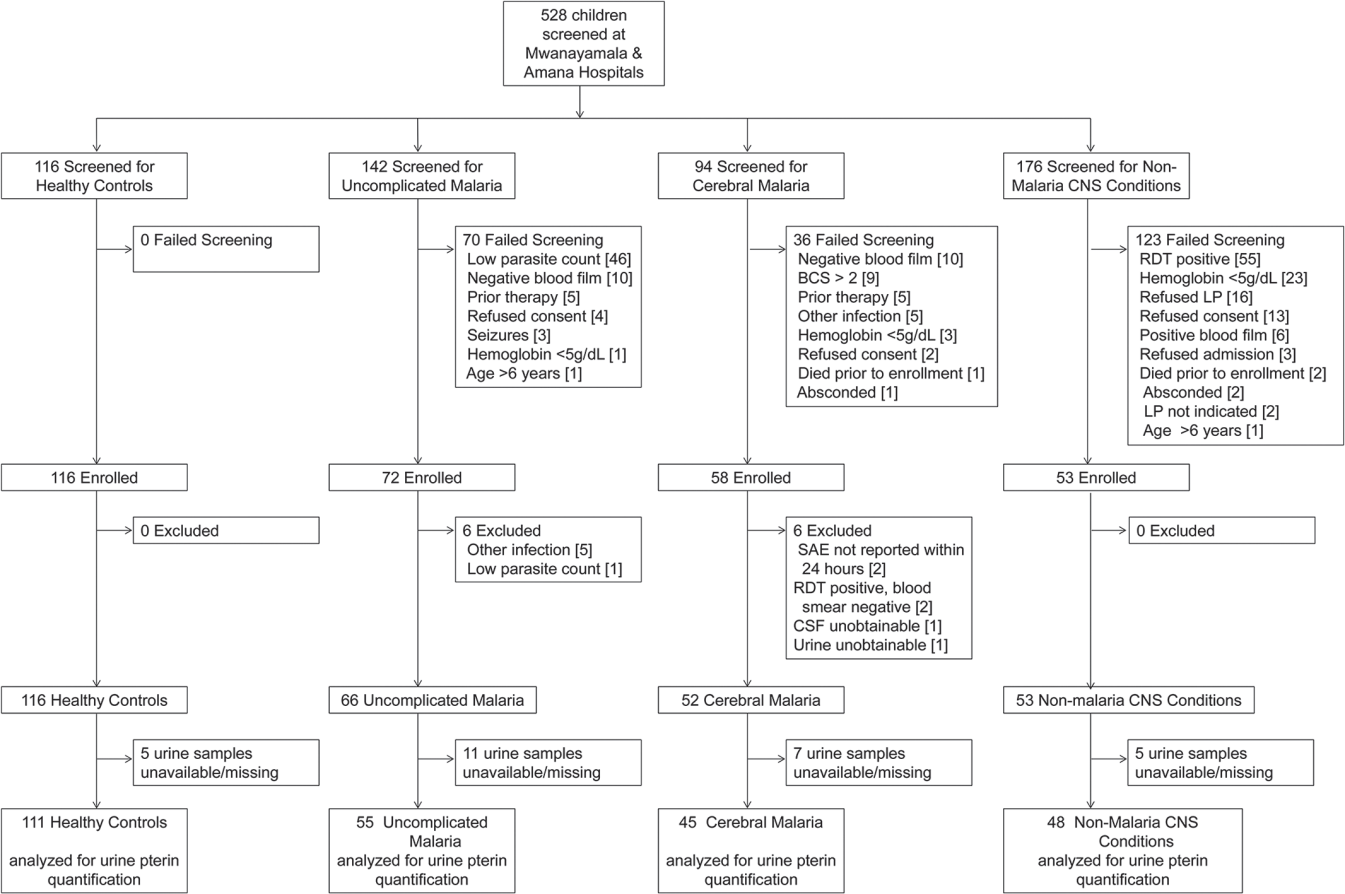
Study flow diagram. Screening, enrollment and *post-hoc* exclusions for the four clinical groups are depicted. CNS = central nervous system; BCS = Blantyre Coma Score; RDT = rapid diagnostic test for *P*. *falciparum* (ParacheckPf, Omega Diagnostics); LP = lumbar puncture; SAE = serious adverse event; CSF = cerebrospinal fluid.

From November 2007 to January 2012 we screened 528 children for enrollment into one of four study groups. After exclusions, 66 children with uncomplicated malaria (UM), 52 with cerebral malaria (CM), 53 with non-malaria central nervous system conditions (NMC), and 116 healthy controls (HC) were enrolled (**Fig. 1**). Baseline characteristics comparing the four groups are shown in **Table 1**. Children with CM were significantly older than children from the other groups when compared across all groups and in pairwise comparisons between groups. Two children with CM and one child with NMC had overt renal failure as evidenced by plasma creatinine measurements of 2.7, 5.1, and 2.5 mg/dL, respectively. Children with CM had significantly lower peripheral blood parasitemia, but significantly higher plasma *P*. *falciparum* histidine-rich protein-2 (PfHRP-2) concentrations compared to children with UM. To assess biopterin status in CM compared to other infectious and non-infectious central nervous system (CNS) conditions, we enrolled a comparison group comprised of children presenting to the hospital with NMC for which lumbar puncture was clinically indicated. The NMC group proved to be heterogeneous as expected. Clinical and laboratory investigations indicated that at least 10 of the 53 children with NMC suffered from bacterial or fungal meningitis. No evidence for viral encephalitis, trauma, subarachnoid hemorrhage or metabolic encephalopathy was found. Toxic encephalopathy was possible in one child. Idiopathic seizures and aseptic meningitis were other possible diagnostic categories. Seven children with CM and six children with NMC died while hospitalized. All children with UM recovered.

**Table 1:** Baseline characteristics of the 4 clinical groups.

Variable	Healthy Controls (n = 116)	Uncomplicated Malaria (n = 66)	Cerebral Malaria (n = 52)	Non-Malaria CNS Conditions (n = 53)	p-value
**Age, months**	34 (31–38)	44 (39–49)	51 (47–55)	29 (23–34)	<0.001
**Male sex, no. (%)**	65 (56.5)	35 (53.0)	34 (65.4)	29 (55.8)	0.556^A^
**Weight, kg**	12.9 (12.2–13.6)	14.3 (13.4–15.2)	15.4 (14.4–16.4)	11.3 (10.3–12.3)	<0.001
**Axillary temperature,°C**	36.7 (36.6–36.7)	39 (38.8–39.3)	38.3 (38.0–38.6)	37.7 (37.4–38.1)	<0.001
**Respiratory rate**	32 (31–33)	36 (34–38)	43 (40–47)	37 (34–41)	<0.001
**Last meal, hours**	3.2 (2.9–3.6)	5.9 (4.7–7.1)	5.9 (4.1–7.7)	3.8 (2.6–4.9)	<0.001
**Hemoglobin, g/dL**	10.8 (10.4–11.1)	8.4 (7.8–9.0)	7.0 (6.5–7.5)	9.8 (9.3–10.3)	<0.001
**Blood glucose, mg/dL** ^**C**^	N/D	119 (96–143)	119 (105–132)	119 (93–147)	0.996
**Plasma bicarbonate, mmol/L** ^**C**^	N/D	19.1 (16.1–22.1)	20 (18.8–21.3)	19 (17–21)	0.678
**Plasma creatinine, mg/dL** ^**C**^	N/D	0.3 (0.3–0.4)	0.8 (0.5–1.0)	0.5 (0.4–0.7)	0.031
**Urine leukocyte esterase present, no. (%)** ^**D**^	3 (5.8)	7 (19.4)	4 (8.5)	2 (4.3)	0.08^A^
**Plasmodium falciparum parasite density, x 10^3^/ μL; median (interquartile range)**	0	87,055 (30,115–209,550)	45,231 (9396–128,231)	0	0.026^B^
**Plasma PfHRP-2, ng/mL; median (interquartile range)**	0	517.4 (39.3–1536.0)	1,086.5 (379.1–2,585.5)	0	0.011^B^

Data mean (95% confidence interval values) unless otherwise indicated.

ANOVA with Bonferroni post-estimate unless otherwise indicated.

A = Chi-squared,

B = Wilcoxon Rank Sum.

PfHRP-2 = *Plasmodium falciparum* histidine-rich protein-2.

N/D = Test not done.

C = We were unable to obtain measurements of all analytes for all patients enrolled.

Glucose measurements were obtained in 27, 48 and 43 of the UM, CM and NMC children, respectively.

Bicarbonate results were obtained 31, 48 and 47 of the UM, CM, and NMC children, respectively. Creatinine results were obtained in 16, 39 and 40 of the UM, CM and NMC children, respectively.

^D^ = Three children (1 HC, 1 UM and 1 CM) with bacteriuria remained in the study as the urine was collected by bag collection (i.e., not collected via sterile, invasive procedures), and there was no clinical suspicion of urinary tract infection in any of the three subjects.

### Abnormal distribution of urine biopterins in children with cerebral malaria

**Fig 2:**
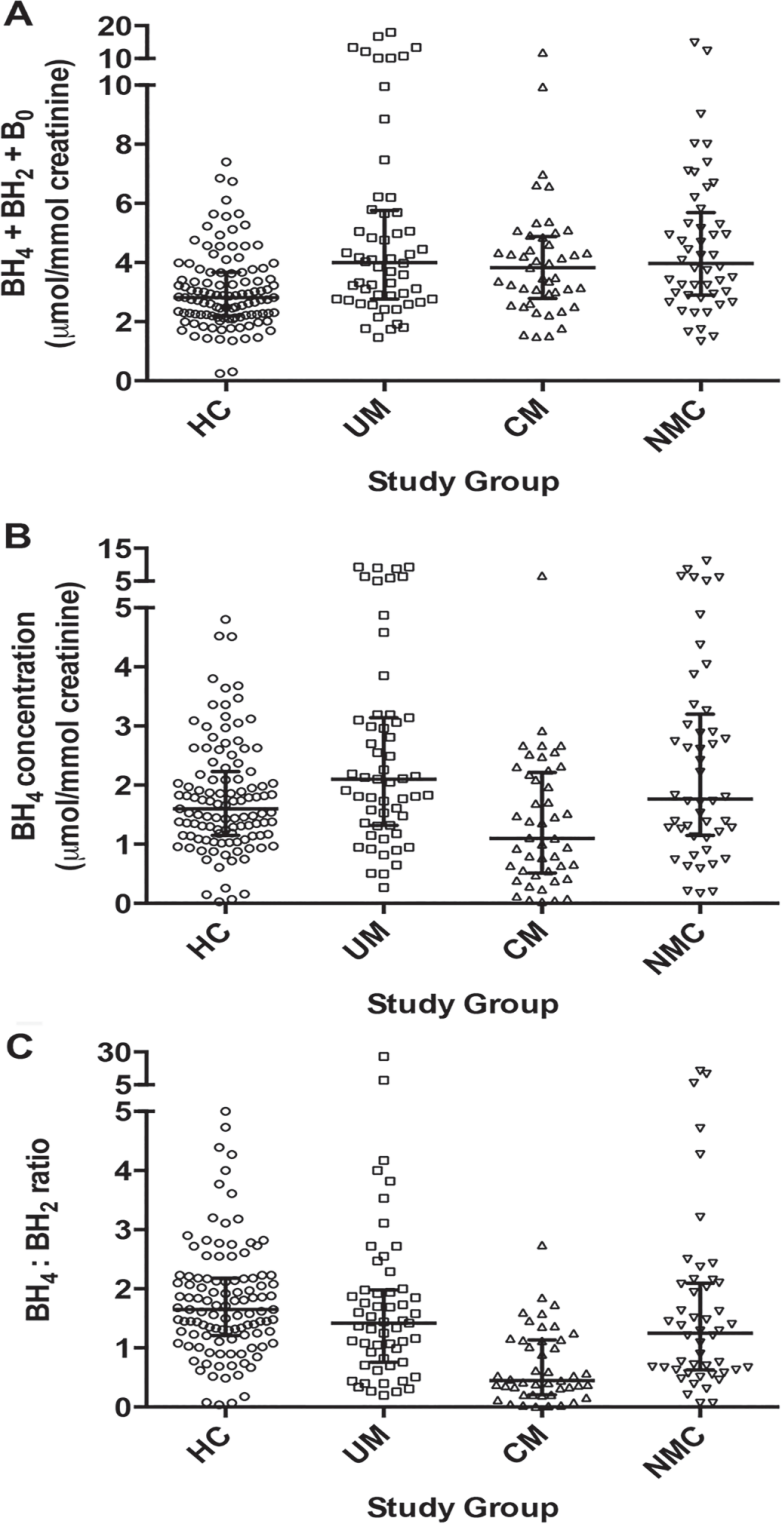
Urine biopterin concentrations at enrollment in the 4 clinical groups. **A**: Comparison of total urine biopterins (tetrahydrobiopterin [BH4] + dihydrobiopterin [BH2] + biopterin [B0]) concentrations at enrollment for healthy controls (HC, n = 111), uncomplicated malaria (UM, n = 55), cerebral malaria (CM, n = 45) and non-malaria central nervous system conditions (NMC, n = 48) (p<0.001 by Kruskal-Wallis). **B**: urine tetrahydrobiopterin (BH_4_) concentrations at enrollment for all 4 groups (p<0.001 by Kruskal-Wallis). Central line indicates median. Upper and lower lines indicate inter-quartile range. **C**: Comparison of urine tetrahydrobiopterin to dihydrobiopterin (BH4:BH2) ratio at enrollment for all 4 groups (p<0.001 by Kruskal-Wallis). The number of subjects with a sample measured in **[B]** and **[C]** is the same as Noted above in **[A]**. The central line indicates median. Upper and lower lines indicate interquartile range.

Urine pterin concentrations differed significantly across the four groups (**Table 2**). Compared to HC (n = 111), total biopterin levels (BH_4_ + BH_2_ + B_0_) were increased in UM (n = 55) (p<0.001), CM (n = 45) (p<0.001), and NMC (n = 48) (p<0.001) (**Fig. 2, panel A**). Urine BH_4_ concentrations were significantly lower in CM than in each of the other three groups (p≤0.005 for all pairwise comparisons) (**Fig. 2, panel B**). The statistical significance persisted in a linear regression model to control for plasma creatinine (p = 0.021) and in a linear regression model to control for age, weight, gender and plasma creatinine (p = 0.038). Using logistic regression, we found no significant co-variation of urine BH_4_ by presence or absence of pyuria (OR 1.21 [0.65–2.25]; p = 0.56) or by presence or absence of bacteriuria (OR 0.70 [0.34–1.46]; p = 0.34). Urine BH_2_ was significantly higher in CM compared to each of the other three groups (p<0.05 for all comparisons). Urine BH_4_ values differed significantly when compared across UM (2.10 [IQR 1.32–3.14]), CM survivors (1.10 [IQR 0.48–2.25] μmol/mmol creatinine) and CM fatalities (1.02 [IQR 0.77–2.09] μmol/mmol creatinine) (p<0.001).

**Table 2:** Urine pterins and plasma phenylalanine measurements at enrollment in the 4 clinical groups.

Variable	Healthy Controls (n = 111)	Uncomplicated Malaria (n = 55)	Cerebral Malaria (n = 45)	Non-Malaria CNS Conditions (n = 48)	p-value
**BH_4_** ^**A**^ ^,^ ^**B**^ ^,^ ^**C**^	1.60 (1.15–2.23)	2.10 (1.32–3.14)	1.10 (0.55–2.18)	1.52 (1.01–2.71)	<0.001
**BH_2_** ^**A**^ ^,^ ^**B**^ ^,^ ^**C**^	0.96 (0.75–1.33)	1.60 (0.95–2.98)	2.24 (1.55–2.89)	1.83 (1.13–2.30)	<0.001
**Biopterin** ^**A**^	0.09 (0.05–0.16)	0.24 (0.12–0.52)	0.22 (0.08–0.37)	0.13 (0.08–0.30)	<0.001
**Total Biopterins** ^**A**^	2.82 (2.19–3.67)	4.03 (2.77–5.79)	3.83 (2.95–4.85)	3.97 (2.91–5.57)	<0.001
**BH_4_/BH_2_ Ratio** ^**A**^ ^,^ ^**B**^ ^,^ ^**C**^	1.64 (1.21–2.18)	1.42 (0.75–1.98)	0.45 (0.21–1.13)	1.25 (0.63–2.09)	<0.001
**BH_4_/(BH_2_+B_0_)** ^**A**^ ^,^ ^**B**^ ^,^ ^**C**^	1.52 (1.04–1.97)	1.17 (0.68–1.85)	0.39 (0.19–1.06)	1.12 (0.54–1.93)	<0.001
**BH_4_/Total Biopterins** ^**A**^ ^,^ ^**B**^ ^,^ ^**C**^	0.60 (0.51–0.66)	0.54 (0.40–0.65)	0.28 (0.16–0.51)	0.53 (0.35–0.66)	<0.001
**NH_2_** ^**A**^ ^,^ ^**B**^	3.20 (1.98–4.69)	18.36 (11.62–24.37)	19.86 (15.86–23.22)	6.62 (3.69–11.77)	<0.001
**N_0_** ^**A**^ ^,^ ^**B**^ ^,^ ^**C**^	0.74 (0.52–1.16)	3.93 (2.26–3.07)	5.48 (4.11–6.43)	1.43 (0.88–3.07)	<0.001
**Total Neopterin** ^**A**^ ^,^ ^**B**^	3.89 (2.55–5.90)	22.39 (14.02–32.22)	25.26 (20.21–30.58)	7.96 (4.53–14.85)	<0.001
**NH_2_/N_0_ Ratio** ^**A**^ ^,^ ^**C**^	4.39 (3.26–5.52)	4.72 (3.52–5.57)	3.74 (3.22–4.22)	5.19 (2.83–9.61)	0.018
**Phenylalanine** ^**A**^ ^,^ ^**B**^ ^,^ ^**D**^	53 (45–60)	101 (80–165)	129.5 (91–208)	75 (63–114)	<0.001

Units: urine pterins μmol/mmol creatinine; amino acid μM.

Median and inter-quartile ranges, p-value Kruskal-Wallis comparison across all 4 groups. BH_4_ = tetrahydrobiopterin, BH_2_ = dihydrobiopterin, B_0_ = biopterin, NH_2_ = dihydroneopterin, N_0_ = neopterin.

A = p-value <0.05 by Wilcoxon Rank Sum comparison between cerebral malaria and healthy controls

B = p-value < 0.05 by Wilcoxon Rank Sum comparison between cerebral malaria and non-malaria CNS condition

C = p-value < 0.05 by Wilcoxon Rank Sum comparison between cerebral malaria and uncomplicated malaria

D = Number of plasma samples for healthy controls, uncomplicated malaria, cerebral malaria and non-malaria CNS conditions was 109, 61, 50, and 51, respectively.

NOS activity is affected not only by the availability of the cofactor BH_4_, but also by the stoichiometric balance of BH_4_ relative to its major oxidized metabolite BH_2_. BH_2_ competes with BH_4_ at the NOS binding site and can directly inhibit NOS activity [36]. The BH_4_:BH_2_ ratio has been proposed as the critical determinant of NO synthesis by endothelial NOS (eNOS) [37]. Accordingly, we analyzed this ratio in the four clinical groups enrolled. The BH_4_:BH_2_ ratio was significantly lower in CM compared to each of the other groups (p<0.001 for all comparisons) (**Fig. 2, panel C**). In our a priori analytical plan, we also compared the ratio of reduced to oxidized biopterins (BH_4_: BH_2_+B_0_) and the proportion of total biopterins as BH_4_ (BH_4_/ BH_4_+ BH_2_+ B_0_). Using these alternative renderings to examine reduced to oxidized biopterins, we found the same statistical relationships between CM in pairwise comparison to the other three clinical groups (Table 2).

Compared to children with likely bacterial or cryptococcal meningitis (n = 10), children with cerebral malaria had lower BH_4_ than this subset of NMC children (1.10 [IQR 0.55–2.18] vs. 1.63 [IQR 1.39–2.90] μmol/mmol creatinine; p = 0.06), significantly higher BH_2_ than children in this subset (2.24 [IQR 1.55–2.89] vs. 1.50 [IQR 0.93–2.27] μmol/mmol creatinine; p = 0.05), and a significantly lower BH_4_:BH_2_ ratio than children with likely bacterial or cryptococcal meningitis (0.45 [0.21–1.13] vs. 1.35 [IQR 0.70–3.21]; p = 0.002).

Collectively, these measurements of urine biopterins demonstrate deficiency in systemic BH_4_ in CM.

### Elevated urine neopterins in cerebral malaria

Guanosine triphosphate cyclohydrolase I (GTPCH-1) is the rate-limiting enzyme in the first step for BH_4_ *de novo* synthesis. Mononuclear phagocytes activated by pro-inflammatory cytokines (e.g., INF-γ, TNF-α) show enhanced transcription of the GTPCH-1 gene, *GCH1* [38]. However, the activity of the second enzyme for biopterin synthesis, 6-pyruvoyl tetrahydropterin synthase (PTPS), is constitutively very low in these cells and is unresponsive to cytokine-induced transcription activation [28,39,40]. The result is shunting of the GTPCH-1 product, 7,8 dihydroneopterin triphosphate, to neopterin end products—dihydrobiopterin (NH_2_) and its oxidized metabolite, neopterin (N_0_) [41]. These neopterin end products accumulate within mononuclear phagocytes and then exit the cells to be excreted in urine. Although the biological function of the neopterins remains obscure, elevated total urine neopterin (NH_2_ + N_0_) is established as a marker of the cell-mediated immune response in a variety of inflammatory states [42]. NH_2_ measurement relies upon oxidation of NH_2_ to the naturally fluorescent N_0_ *in vitro* for quantification [43]. We took advantage of our collection method for preserving urine neopterins in their *in vivo* reduced (NH_2_) and oxidized (N_0_) states to quantify them in CM. In doing so, we sought to determine whether the neopterins, like the biopterins, have a redox ratio skewed towards oxidation in children with CM.

**Fig 3:**
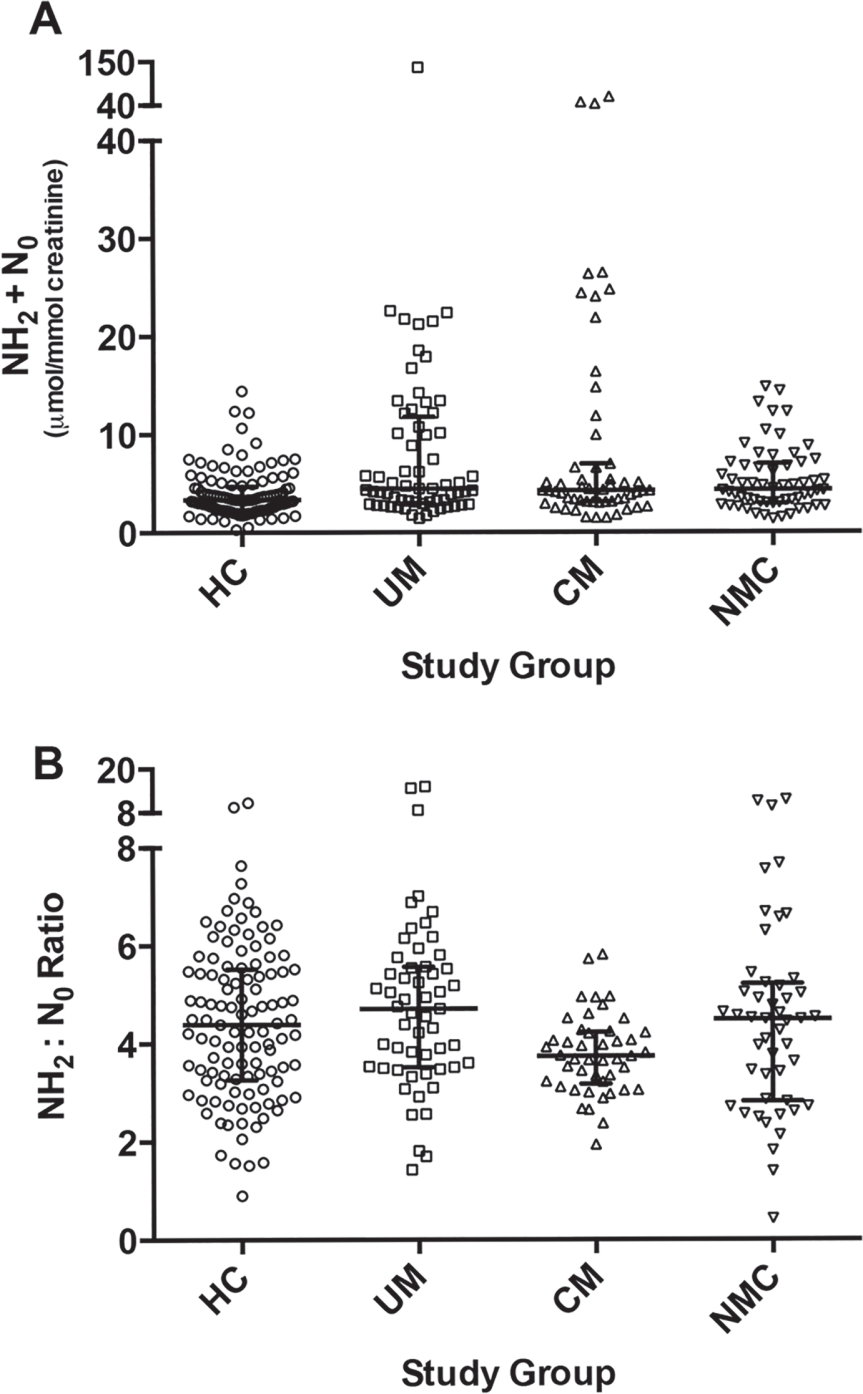
Urine neopterin concentrations at enrollment in the 4 clinical groups. **A**: Comparison of total urine neopterins, dihydroneopterin plus neopterin (NH_2_ + N_0_), concentrations at enrollment for healthy controls (HC, n = 111), uncomplicated malaria (UM, n = 55), cerebral malaria (CM, n = 45) and non-malaria central nervous system conditions (NMC, n = 48) (p<0.001 by Kruskal-Wallis). The central line indicates median. Upper and lower lines indicate inter-quartile range. **B**: Comparison of urine dihydroneopterin to neopterin (NH_2_:N_0_) at enrollment for all 4 groups (p = 0.018 by Kruskal-Wallis). The number of subjects with a sample measured is the same as noted above in [A]. The central line indicates median. Upper and lower lines indicate inter-quartile range.

Concentrations of urine neopterin metabolites differed significantly across the four groups (**Table 2**). Compared to HC, total neopterin (NH_2_ + N_0_) levels were increased in UM, CM and NMC (p<0.001 for all comparisons) (**Fig. 3, panel A**). Total neopterin levels did not differ significantly in UM compared to CM (p = 0.113). The ratio of reduced to oxidized neopterins (NH_2_:N_0_) was significantly decreased in CM compared to UM (p = 0.001) and HC (p = 0.017). This ratio did not statistically differ between CM and NMC (p = 0.069) (**Fig. 3, panel B**). The reduced to oxidized ratio for neopterins correlated with the reduced to oxidized ratio for biopterins (BH_4_: BH_2_+B0) (r = 0.28, p<0.001) and oxidized neopterin (N0) correlated with oxidized biopterin (BH_2_) (r = 0.63, p<0.001). We conclude from these measurements that while total urine neopterin levels did not differ in CM compared to UM, quantifying neopterins in their reduced to oxidized states may indicate oxidative stress in CM compared to UM.

### Elevated angiopoietin-2 in cerebral malaria

**Fig 4:**
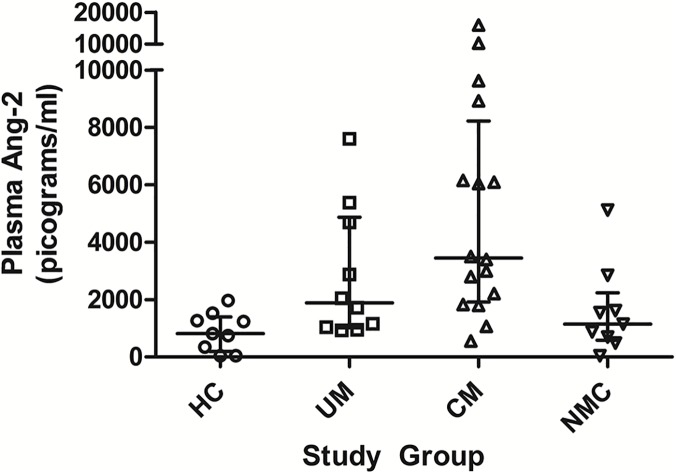
Plasma angiopoietin-2 concentrations at enrollment in the 4 clinical groups. Comparison of plasma angiopoietin-2 (Ang-2) concentrations at enrollment in a subset of healthy controls (HC, n = 9), uncomplicated malaria (UM, n = 10), cerebral malaria (CM, n = 16) and non-malaria central nervous system conditions (NMC, n = 9) (p<0.001 by Kruskal-Wallis). The central line indicates median. Upper and lower lines indicate inter-quartile range.

Angiopoietin-2 (Ang-2) is a ligand released from endothelial Weibel-Palade bodies that acts as an autocrine mediator of endothelial activation [44], including up-regulation of intercellular adhesion molecule (ICAM) receptors on endothelial cells [45]. Ang-2 plasma levels correlate with clinical severity in pediatric sepsis [10,46] and are independently associated with mortality in severe malaria [10,46,47]. NO is a known inhibitor of endothelial Weibel-Palade bodies exocytosis [48]. *In vitro* endothelial cell experiments demonstrate biopterin redox status impacts endothelial dysfunction [37], but it is unknown whether whole body stores of biopterins correlate with *in vivo* circulating NO-dependent mediators of endothelial activation. To assess for associations between systemic biopterin status and endothelial activation we measured Ang-2 in a subset of randomly selected children enrolled as HC (n = 9), UM (n = 10), CM (n = 16), and NMC (n = 9). Plasma Ang-2 concentration differed significantly across the four groups (**Fig. 4**)—HC (815 [IQR 345–1264] pg/mL), UM (1882 [IQR 1035–4693] pg/mL), CM (3445 (IQR 2014–7534 pg/mL), and NMC (1149 [691–1616] pg/mL) (p<0.001). Pairwise comparisons among groups showed that median plasma Ang-2 levels were higher in CM compared to HC (p<0.001), UM (p = 0.082) and NMC (p = 0.006). Correlations between plasma Ang-2 and urine biopterin metabolites were assessed within each clinical group and no significant correlations were noted. A correlation scatterplot of plasma Ang-2 and urine BH_2_ values does visually demonstrate a non-significant correlation (S1 Fig.), indicating that the small sample size may have limited our ability to establish a significant correlation. We conclude that Ang-2 is significantly elevated in severe pediatric falciparum malaria, but we were unable to demonstrate a correlation between Ang-2 and urine biopterin metabolites, possibly due to small sample size.

### Peripheral blood mononuclear cell mRNA for guanosine triphosphate cyclohydrolase I in children with malaria

To determine if the low urine levels of BH_4_ observed in CM could in part be explained by decreased *GCH1* expression for GTPCH-1, the rate-limiting enzyme in pterin biosynthesis [29], we measured *GCH1* mRNA in peripheral blood mononuclear cells (PBMC) isolated from a randomly selected sub-set of children enrolled as HC (n = 5), UM (n = 8) and CM (n = 9). *GCH1* mRNA transcription was at least as high in each malaria group [UM (1.35 [IQR 1.11–1.97] 2^-ddCt^) and CM (1.39 [IQR 0.88–1.77] 2^-ddCt^)] relative to controls (0.82 [IQR 0.60–0.84] 2^-ddCt^) (p = 0.082). We also measured *GCH1* mRNA in children enrolled into a separate cohort using the same inclusion/exclusion criteria as HC (n = 33) and as CM (n = 9). Combining the measurements from both cohorts [HC n = 38 and CM n = 18], we found no significant difference in *GCH1* mRNA between these two groups. We conclude that PBMC *GCH1* mRNA and pterin synthesis is not decreased in children with malaria and does not account for the lower BH_4_.

### Hyperphenylalaninemia in children with uncomplicated malaria and cerebral malaria

We measured plasma phenylalanine in all 4 clinical groups to confirm our previous observation of hyperphenylalaninemia in clinical malaria [18] and to assess the relationship between systemic BH_4_ status and plasma phenylalanine concentration. Hyperphenylalaninemia (upper limit of normal in children age > 12 months, 80uM; upper limit of normal in children age < 12 months, 100 uM) was found in 4 (3.7%) of 109 HC, 44 (72.1%) of 61 UM, 40 (80%) of 50 CM, and 23 (45.1%) of 51 NMC. Plasma phenylalanine concentrations differed significantly across all groups (**Table 2**). This association remained after using linear regression to control for age, weight, and fasting duration. Using the same linear regression model, plasma phenylalanine concentrations differed significantly in CM compared to UM (p = 0.04), and across UM, CM survivors and CM fatalities (p = 0.015). Plasma phenylalanine concentration correlated with urine BH_2_ concentration among HC (r = 0.29; p = 0.002), UM (r = 0.43; p = 0.001), and NMC (r = 0.32; p = 0.03), but among CM the correlation was non-significant (r = 0.22; p = 0.15). Similarly, when analyzing UM and CM children jointly, there was a significant correlation between plasma phenylalanine and BH_2_ was observed (r = 0.38; p<0.001), but we when employed a partial correlation test to control for malaria disease severity, this correlation was non-significant (r = 0.17; p = 0.09). Plasma phenylalanine did not correlate with BH_4_ or the BH_4_/BH_2_ ratio. Despite a non-significant correlation among CM, overall we found a moderate correlation between plasma phenylalanine and urine BH_2_ levels, a result that is consistent with the body of literature demonstrating the ability of BH_2_ to inhibit PAH enzymatic conversion of phenylalanine to tyrosine [28].

### Bivariate and multivariate regression analysis correlating urine BH_4_ to severity of malaria

In bivariate logistic regression among all malaria patients (**Table 3**), a unit decrease in Log_10_-urine BH_4_ and Log_10_-urine BH_4_/BH_2_ ratio was associated with 2.94-fold (95% CI 1.65–5.23) increase and 3.29-fold (95% CI 1.87–5.76) increase, respectively, in the odds of CM (p<0.001). A unit increase in log_10_-urine BH_2_ was associated with a 1.91-fold increase (95% CI 0.99–3.67) in the odds of CM (p = 0.04). In the backward stepwise multivariate logistic regression model for risk of CM, variables were retained in the model if their p-value was < 0.2. The final model included urine BH_4_, urine BH_2_, *P*. *falciparum* histidine-rich protein-2 (PfHRP-2), respiratory rate and weight (Table 3). Urine BH_4_ and urine BH_2_ levels were independently associated with odds of CM: a unit decrease in Log_10_-urine BH_4_ was associated with 3.85-fold (95% CI 1.89–7.61) increase in the odds of CM (p<0.001). Note that as Table 3 models risk of CM for every unit increase in a given variable, the inverse is displayed—i.e., a unit increase in Log_10_-urine BH_4_ was associated with 0.26-fold (95% CI 0.13–0.53) decrease in odds of CM [p<0.001]. A unit increase in Log_10_-urine BH_2_ was associated with a 2.86-fold (95% CI 1.01–8.07) increase in the odds of CM (p = 0.047).

**Table 3:** Bivariate and multivariate logistic regression analysis of factors associated with increased odds of cerebral malaria.

Variable	N	Bivariate OR (95% CI)	p-value	Multivariate OR (95% CI)	p-value
**Urine BH_4_**	100	0.34 (0.19–0.60)	<0.001	0.26 (0.13–0.51)	<0.001
**Urine BH_2_**	100	1.91 (0.99–3.68)	0.053	3.38 (1.24–9.21)	0.018
**Phenylalanine**	111	1.78 (0.89–3.55)	0.101	—	>0.05^A^
**PfHRP-2**	106	1.26 (1.03–1.53)	0.024	—	>0.05
**Hemoglobin**	116	0.73 (0.60–0.89)	0.001	—	>0.05^A^
**Bicarbonate**	79	1.03 (0.95–1.11)	0.480	—	—^B^
**Respiratory Rate**	118	1.07 (1.04–1.17)	0.001	1.10 (1.05–1.17)	<0.001
**Weight**	118	1.09 (0.98–1.21)	0.106	1.19 (1.02–1.39)	0.026
**Age**	118	1.02 (1.00–1.05)	0.038	—	>0.05^A^
**Sex**	118	1.67 (0.79–3.54)	0.181	—	—^B^
**Last meal**	118	0.99 (0.94–1.07)	0.99	—	>0.05^A^

CI = confidence intervals

BH_4_ = tetrahydrobiopterin

BH_2_ = dihydrobiopterin

PfHRP-2 = *P*. *falciparum* histidine-rich protein-2

Note: Phenylalanine, Urine BH_4_, Urine BH_2_ and PfHRP-2 were log-transformed to enable regression analysis. Number of observations included in multivariate model was 96.

A = included in multivariate logistic regression analysis, but not retained in the final model due to p-value > 0.05 in multivariate analysis.

B = not included in multivariate logistic regression analysis because of non-significance in bivariate logistic regression analysis.

The urine BH_4_:BH_2_ ratio is not included in the logistic regression model due to co-linearity with BH_4_. When the multivariate model included the BH_4_:BH_2_ ratio *in lieu* of urine BH_4_, the odds ratio of CM was comparable to the odds ratio of CM seen with urine BH_4_: a unit decrease in Log_10_-urine BH_4_:BH_2_ ratio was associated with a 3.63-fold (95% CI 1.95–6.72) increase in the odds of CM (p<0.001).

## Discussion

Systemic BH_4_ concentrations are significantly lower in children with CM compared to HC, UM, and NMC. BH_4_ concentrations were likewise significantly lower in fatal CM when compared across the spectrum of UM and CM survivors, and a decrease in urine BH_4_ was independently associated with a 3.85-fold increase in odds of CM. While two prior studies reported conflicting results on BH_4_ levels in cerebrospinal fluid [17,49], our study is the first published analysis of systemic biopterin levels in severe human malaria. Our findings have important implications regarding the underlying reasons for the decreased NO bioavailability and microvascular dysfunction observed in severe malaria [16,19,21]. These data also represent a novel link between the extensive vascular medicine research regarding the effects of BH_4_ and BH_2_ on the endothelium [27] and the vasculopathic mechanisms of severe malaria [16].

Our prior studies have demonstrated that NO is important in protecting against severe malaria, including CM, and have demonstrated several mechanisms by which NO bioavailability is decreased in malaria. These mechanisms include: low overall NO production [21]; low NOS2 protein levels in peripheral blood mononuclear cells [21]; low NOS activity in PBMC [22]; NOS2 single nucleotide polymorphisms that modulate NO production [23]; increased cell-free hemoglobin, an NO quencher [50]; decreased NOS substrate arginine [6,24]; and increased asymmetric dimethyl arginine (ADMA) [25,26], an endogenous inhibitor of NOS activity [51]. Our present study documents another potential mechanism for low NO production in severe malaria—diminished levels of NOS cofactor, BH_4_. Our prior studies of NO in malaria have focused on NOS2 (inducible NOS), which is expressed in multiple tissues, including brain and endothelium. Our current findings apply not only to NOS2, but to all three NOS isoforms (endothelial, inducible and neuronal), as BH_4_ is an obligate co-factor for all three NOS isoforms and deficiency of BH_4_ can result in diminished NOS activity and cause uncoupling in all NOS isoforms. We observed that total urine biopterins (which reflect total body biopterins) were increased in mild and severe malaria. Expression of PBMC mRNA for CGH1, the rate-limiting enzyme in biopterin synthesis, was at least as high in malaria as in HC. While pterin synthesis in human leukocytes may not reflect biopterin bioavailability in other tissues (e.g., the endothelium), this finding is consistent with our observation of increased total urine biopterins in malaria. Beyond the absolute totals, we think the more significant observation is the markedly skewed distribution of biopterin species in CM cases—we found that nearly 2/3 of biopterins were oxidized to inactive forms, a finding not previously reported. Tanzanian children with CM had significantly lower urine BH_4_ concentrations and a lower BH_4_:BH_2_ ratio compared to HC, UM and NMC.

Quantification of the different redox states of biopterins in malaria—fully reduced, BH_4_, and its oxidized metabolites, BH_2_ and B_0_—may be a novel method for measuring oxidative stress in severe malaria and possibly other critical illnesses. The same rationale applies to the NH_2_:N_0_ ratio. Our finding of increased levels of neopterin in UM and CM is consistent with prior reports of neopterin concentrations in malaria [52,53]. While the pathophysiologic role of neopterin remains unclear, the ratio of reduced to oxidized neopterin (NH_2_:N_0_) might represent a novel parameter to quantify mononuclear phagocyte redox state. The decrease in both BH_4_:BH_2_ and NH_2_:N_0_ ratios indicates increased systemic oxidative stress in CM.

As a NOS co-factor, BH_4_ performs both structural and biochemical functions. It stabilizes the NOS homodimer, thereby enabling NOS catalytic function, and it donates an electron to form a transient BH_4_∙^+^ radical which is required for oxidation of L-arginine to L-citrulline [27]. However, in the absence of BH_4_, NOS catalyzes a reaction in which NADPH-derived electrons reduce heme iron thereby binding and activating oxygen [54]. Without BH_4_-dependent L-arginine oxidation, activated oxygen is released from the heme catalytic center of NOS as superoxide. This generation of superoxide is termed NOS “uncoupling,” as L-arginine oxidation to L-citrulline is no longer coupled to oxygen activation for NO synthesis and release [27]. In an environment where both superoxide (uncoupled NOS) and NO (coupled NOS) are generated, both products may react to yield peroxynitrite [55,56]. Peroxynitrite and superoxide induce further uncoupling by oxidizing available BH_4_ to BH_2_ [57], thereby decreasing the availability of BH_4_ for NO synthesis. This uncoupling is compounded by BH_2_ accumulation, which competes with BH_4_ at its NOS binding site, further inhibiting NOS-catalyzed NO synthesis [37]. Thus a feed-forward mechanism, initiated by low intracellular BH_4_ levels, ensues and leads to oxidative injury caused by superoxide and peroxynitrite [58]. Experimental models of eNOS activity indicate that the BH_4_:BH_2_ ratio, which we found to be significantly decreased in CM compared to all other groups, is the measurement that most strongly correlates with NOS uncoupling [37].The hyperphenylalaninemia that we previously described in UM and CM [18] was one of the initial observations that led us to test the hypothesis of impaired BH_4_ in CM, since BH_4_ is an essential cofactor for the enzymatic hydroxylation of phenylalanine to tyrosine. We confirmed our prior finding of elevated plasma phenylalanine in UM and CM. Our present findings suggest that this elevation in malaria is secondary to decreased systemic concentrations of BH_4_ and increased concentrations of BH_2_.

Decreased BH_4_ and increased BH_2_ systemic levels in children with CM may be relevant to the central pathogenic mechanisms in severe malaria—endothelial activation and dysfunction and the sequestration of pRBCs, unparasitized RBCs, mononuclear cells, and platelets resulting in microcirculatory congestion of post-capillary venules [14,16,19,59]. Endothelial cells low in intracellular BH_4_ and high in BH_2_ favor decreased NO production by NOS, and low NO synthesis is associated with endothelial dysfunction [6]. The effect of decreased NO production due to NOS uncoupling may exacerbate microvascular sequestration in several ways. Low NO states are associated with decreased RBC deformability [60]. *In vitro* endothelial cell models have shown that increased bioavailability of NO is associated with decreased cytoadherence of *P*. *falciparum*-infected RBCs via down-regulation of endothelial receptors for *P*. *falciparum* erythrocyte membrane protein [61,62]. NO deficiency causes increased vascular tone with decreased vessel diameter and possible flow impedance. Additionally, NOS uncoupling due to low BH_4_ and high BH_2_ would also favor generation of peroxynitrite, a highly reactive species thought to exacerbate endothelial cell dysfunction [63]. Given that low BH_4_ levels may be contributing to endothelial dysfunction and sequestration in severe malaria, adjunctive therapy to reduce BH_2_ and regenerate BH_4_ warrants further study [64,65]. The need for such studies is also supported by an experimental animal model of cerebral malaria demonstrating that the blunted cerebral arteriolar response to NOS agonists is partially recovered by supplementation with BH_4_ [66].

While our study has many strengths, including rigorous case definitions for the four clinical groups and rigorous methods for sample collection, processing and analysis, we acknowledge several limitations. The specificity of the WHO case definition for CM may be as low as 77% [13]. The causes and severity of illness in the NMC group were heterogeneous, making comparisons with this group more tenuous. Since we were able to measure plasma creatinine in only 16 UM and 39 CM patients, we did not include plasma creatinine in the final multivariate logistic regression model assessing for an independent association between urine BH_4_ concentrations and malaria severity. We therefore cannot exclude that the significant association between BH_4_ and malaria severity is dependent upon renal function. We performed a multivariate logistic regression sub-model for the 48 malaria patients who had urine biopterin and plasma creatinine results. In this sub-model we found that only respiratory rate (OR 1.11 [95% CI 1.01–1.21]; p = 0.04) and plasma creatinine (OR 201.42 {95% CI 8.15–4978.96]; p = 0.001) were independently associated with odds of cerebral malaria (BH_4_ OR 0.34 [95% CI 0.09–1.28;p = 0.11). This indicates that plasma creatinine has a statistically significant association with malaria severity, but the wide confidence intervals show a lack of power for more precisely assessing this relationship and its impact on the association between urine BH_4_ and malaria severity. While the lack of plasma creatinine results is a key limitation of our final multivariate logistic regression model, we note the following in regards to the urine BH_4_ measurements and renal function: 1) only two children (both with CM) had clinically significant renal impairment; 2) A linear regression model of urine BH_4_ showed no significant variance by plasma creatinine among the 85 children in whom both a urine BH_4_ measurement and a plasma creatinine measurement. 3) Total urine biopterins were increased in CM patients, which argues against the higher mean plasma creatinine values in CM as accounting for the decreased levels of BH_4_ in the urine of CM patients. Inclusion of plasma creatinine in the model would have been optimal, but the reasons detailed above, we think exclusion of creatinine from the multivariate logistic regression model is justified and preferred.

Additional limitations include the fact that we did not measure NO bioavailability directly and that our findings do not demonstrate a causal link between endothelial cell dysfunction and low systemic BH_4_. Unlike our companion study demonstrating decreased systemic BH_4_ in Indonesian adults with severe falciparum malaria, we do not have measures of endothelial function in this study to compare with the decreased levels of systemic BH_4_ we have observed in Tanzanian children with CM. Among a subset of the cohort we were able to measure Ang-2, a mediator of endothelial cell activation that is associated with reduced NO-bioavailability in both severe malaria and sepsis [10,67]. The markedly elevated plasma Ang-2 levels and the abnormal urine biopterin metabolite levels observed among children with malaria were not significantly correlated. A small sample size may have limited our ability to show a significant correlation between Ang-2 and BH_2_. While we speculate that the highly perturbed BH_4_: BH_2_ ratio is due to oxidative stress, we do not have additional purported measures of oxidative stress with which to compare biopterin redox status. An alternative explanation for this the low BH_4_: BH_2_ ratio is impaired recycling via DHFR conversion of BH_2_ to BH_4_.

In conclusion, we found that BH_4_, an essential cofactor for NO production, is low in children with CM, and that low BH_4_ concentrations are independently associated with disease severity in children with malaria. Low BH_4_ may contribute to the pathogenesis of severe malaria as it represents an important mechanism of low NO bioavailability. Furthermore, low BH_4_ together with elevated BH_2_ likely leads to generation of reactive oxygen species. These potential sequelae of low systemic BH_4_ likely contribute to endothelial dysfunction and pRBC sequestration—hallmark pathophysiologic features of severe malaria. Interventions that replenish BH_4_ or redistribute the BH_4_:BH_2_ ratio toward reduced BH_4_ merit investigation as adjunctive therapies to improve outcomes in pediatric severe falciparum malaria.

## Materials and Methods

### Study design and site

We conducted a prospective observational study in Dar es Salaam, Tanzania. Children were enrolled from the clinics and the inpatient wards of Amana and Mwanayamala District Hospitals into the following four groups: UM, CM, NMC (see criteria below), and HC. These two district hospitals are separated by 6.5 kilometers, and both are located in semi-urban areas of Dar es Salaam. Once enrolled, children with CM or with NMC were transferred to the clinical research unit at the Hubert Kairuki Memorial University Hospital for further evaluation and care. Approval for this study was obtained from the institutional review boards of Hubert Kairuki Memorial University, United Republic of Tanzania National Medical Research Institute, University of Utah, and Duke University. Informed consent was obtained from parents or guardians of all children, and the U.S. Department of Health and Human Services guidelines for human subjects research were followed.

### Enrollment criteria

Children were 6 months to 6 years in age. The World Health Organization (WHO) case definition for CM was used as the inclusion criteria for the CM cohort: any level of *P*. *falciparum* parasitemia on peripheral blood smear; unarousable coma (Blantyre Coma Score ≤ 2) not attributable to hypoglycemia (blood glucose level < 40 mg/dL) and persisting more than 60 minutes after any convulsion; no other identifiable cause of coma [68]. Inclusion criteria for UM were as follows: a clinical syndrome consistent with malaria and a documented fever (temperature ≥ 38° C) or history of fever within 48 hours from time of enrollment; *P*. *falciparum* parasitemia > 10,000 parasites/μL on Giemsa-stained blood film plus a positive *P*. *falciparum* RDT (Paracheck-Pf; Omega Diagnostics); no other cause of fever identified; no WHO warning signs suggestive of severe disease [68]. These warning signs were the following: inability to suckle, eat and/or drink; excessive vomiting; abnormal respiratory rate or respiratory distress as evidenced by accessory muscle use, suprasternal retractions, or intercostal retractions; recent history of convulsions; altered mental status; inability to sit unaided.

Exclusion criteria for both groups with malaria were any of the following: microscopic evidence of mixed infection with any other *Plasmodium* species; bacterial co-infection as evidenced by septicemia or urinary tract infection; oral or intravenous quinine or oral artemesinin combination therapy initiated > 18 hours prior to enrollment; hemoglobin < 5 mg/dL, as erythrocyte transfusions were not readily available at our study sites. Children with CM were excluded if they had evidence of bacterial meningitis as indicated by isolation of a pathogen from CSF culture or by CSF analysis.

Similar aged healthy children and children presenting with NMC were prospectively enrolled as control groups. The healthy children were enrolled from outpatient well-baby clinics at the two district hospitals. Eligible children had no signs or symptoms of active illness, no febrile illness within the previous two weeks, no history or evidence of an active inflammatory condition, and negative *P*. *falciparum* RDT (Paracheck-Pf; Omega Diagnostics). Children with NMC were eligible if they had a CNS condition for which a lumbar puncture for CSF analysis was clinically indicated as part of diagnostic evaluation and management (e.g. suspected meningitis, encephalitis, hemorrhage, trauma, metabolic, toxic, recurrent seizures as cause for altered mental status). All children enrolled in this group had a Giemsa-stained blood film negative for *Plasmodium* and a negative *P*. *falciparum* RDT (Paracheck-Pf; Omega Diagnostics). A subset of children in this group were classified as likely bacterial or fungal meningitis based on detection of a microbiologic pathogen in CSF and one of the following findings on CSF analysis: white blood cell count > 6/μL, neutrophils present on cytospin Wright stain prep, or glucose < 2/3 plasma glucose level.

### Clinical evaluation and management

At presentation, demographic information, clinical history, and examination were documented using standardized case report forms. Capillary blood samples were obtained for Giemsa-stained thick and thin blood films as well as on-site malaria rapid diagnostic testing. Venous samples for routine laboratory analysis included complete blood count (Beckman-Coulter Act 10), sodium, potassium, chloride, bicarbonate, blood urea nitrogen, creatinine, and venous blood gas (i-STAT 1; Abbott Laboratories). Urine collected for pterin quantification (see below) was also subjected to urine dipstick analysis (Multistix 10 SG; Siemens Healthcare Diagnostics) and urine culture. These blood and urine laboratory results were immediately available to the clinician. Blood cultures were obtained in all children with UM and CM to rule out concomitant bacteremia. Lumbar puncture with opening pressure measurement was done in all patients with coma to evaluate for meningitis. CSF analysis included determination of glucose and protein levels, cell count with differential by trained microscopists, and bacterial and fungal cultures.

Children with UM and CM received anti-malarial therapy and other supportive care as per standard Tanzanian national protocols at the time of the study (artemesinin combined oral therapy and intravenous quinine, respectively). Treatment was initiated as soon as the diagnosis of malaria was suspected. Children with CM and NMC were re-examined daily until death or hospital discharge.

### Urine collection and storage

Urine was collected from subjects at the time of enrollment. Strict adherence to the collection procedure was followed in every case. All collection and handling of urine samples was done in the dark or under dim lighting (collection bags covered with a black, light-impermeable cloth) to prevent photo-oxidation. For quantification of urine biopterins and neopterins, 5–20 ml urine was collected voluntarily into a sterile urine collection cup or with use of a pediatric bag (U-bag urine collector, Hollister Pediatric) affixed to the perineum with adhesive. In both cases, issuing urine was immediately collected into solid dithioerythritol (DTE, approximately 50 mg/ml urine) and diethylene triamine penta-acetic acid (DETAPAC, approximately 5 mg/ml urine) and mixed well to dissolve the powders as described previously [35]. Immediately after collection, urine samples were placed into an insulated transport cooler charged with large cooling packs preconditioned at -80° C. This resulted in freezing of urine samples within minutes. The samples were then stored in a -80° C freezer and then transported to the USA in a liquid nitrogen dry shipper, and subsequently stored at -80° C until pterin analysis. This procedure exceeded in stringency the conditions required to prevent oxidation of reduced pterins previously described [35,69]. Reagent BH_4_ exposed to this procedure was stable at concentrations in the range measured on clinical urine samples; no detectable oxidation of BH_4_ measured by our analytic methods occurred. Conversely, exposure of oxidized reagent pterins to this procedure did not result in any measureable reduction. Thus the analysis of our samples revealed quantities of urine pterins as they existed *in vivo*.

### Quantification of urine pterins

Thawed urine samples were mixed and directly subjected to analysis for BH_4_, BH_2_ and B_0_ and for NH_2_ and N_0_ by high-performance liquid chromatography (HPLC) using sequential electrochemical and fluorescence detection. The procedure relies on reversed phase HPLC separation of pterins, BH_4_, BH_4_, NH_2_, B_0_ and N_0_ in samples of urine. BH_4_ is oxidized to quinonoid dihydrobiopterin (q-BH_2_) and then reduced back to BH_4_. The current generated on the reduction is monitored and used to determine BH_4_ concentration using an ESA Coularray electrochemical integration system. BH_2_ and NH_2_ are oxidized to B_0_ and N_0_ respectively by electrochemistry and then measured by fluorescence using EZ Chrom integration system. B_0_ and N_0_ are not affected by electrochemical electrodes and are measured by their natural fluorescence using the EZ Chrom system. Further details of the analytical methods are as reported previously [70] (see S2 Fig. and see Fig. 2 & 3 in citation 41 for example chromatograms).

### Plasma PfHRP-2 measurement

PfHRP-2, a measure of total parasite biomass, was quantified by ELISA as previously described [6,10] using primary and secondary monoclonal antibodies to *P*. *falciparum* HRP-2 (MPFM-55A and MPFG-55P; Immunology Consultant Laboratories). Concentrations were derived from standard curves utilizing purified PfHRP-2 kindly provided by D. Sullivan (Johns Hopkins University School of Public Health). Samples with ODs outside the linear part of the curve were repeated at an appropriate dilution until an accurate concentration was determined. The lower limit of detection was 1.5 ng/mL.

### Plasma Angiopoietin-2 measurement

Ang-2 was measured by ELISA (R&D Systems) as noted before [10].

### Measurement of mRNA for GTPCH-1 by qRT-PCR

PBMC were isolated using standard methods. RNA was extracted from PBMC and subjected to real-time reverse transcriptase-polymerase chain reaction (RT-PCR) techniques. We used the equivalent of 100 ng RNA per reaction. Stratagene VILO kit was used for the RT reaction, and the Roche faststart universal probe master kit for PCR (Roche Applied Science). Primer/probe sets were designed by the Roche universal probe library application. Primers were then made by IDT while probes were from the Roche universal probe library (http://universalprobelibrary.com) (Roche Applied Science). The quantitative PCR were performed] on an ABI7300 machine. Samples were run in triplicate, or in duplicate if sample amount was limiting. The GAPDH and HPRT1 genes were selected as endogenous control genes. The average Ct of the endogenous controls for each sample was used in the dCt calculation. For individual ddCt values, we used each sample dCt and the averaged healthy control dCt. For group ddCt values, we used the average dCt for each group to calculate ddCt for the group. The data is expressed as 2^-ddCt^, which is the fold change as compared to the average of the healthy controls. **Table 4** displays the relevant sequences for the primers and probes.

**Table 4:** Primers and probes for GTP-cyclohydrolase I mRNA reverse transcriptase polymerase chain reaction.

Gene	Forward Primer	Reverse Primer	Probe number	Probe cat#	Probe sequence
**GTPCH1**	gctgtagcaatcacggaagc	cacctcgcattaccatacaca	67	4688660001	tgctggag
**GAPDH**	agccacatcgctcagacac	gcccaatacgaccaaatcc	60	4688589001	tggggaag
**HPRT1**	tgaccttgatttattttgcatacc	cgagcaagacgttcagtcct	73	4688961001	gctgagga

### Plasma amino acid analysis

Blood samples were collected into heparin tubes, mixed and immediately centrifuged to sediment blood cells. Supernatant plasma was removed to freezing tubes and placed into the transport vessel described above, after which they were stored at -80° C until thawing for analysis. Amino acids were quantified by ion-exchange chromatography with detection using spectrophotometry after reaction with ninhydrin. All amino acid analysis was performed at the Biochemical Genetics Section, ARUP Laboratories, University of Utah in collaboration with Dr. Marzia Pasquale.

### Statistical methods

Statistical analysis was performed with STATA software (version 12.0; StataCorp). Results are presented as mean with 95% confidence intervals for normally distributed continuous variables or median with interquartile range for variables with non-parametric distribution. For continuous variables with normal distribution, differences across groups were compared by ANOVA with Bonferroni adjustment for multiple comparisons and differences between groups were compared using Student’s t-test. For continuous variables with a non-parametric distribution, differences across groups were compared using Kruskal-Wallis test and differences between groups were compared using the Wilcoxon rank-sum test. Correlation between variables with non-parametric distributions was assessed using Spearman correlation coefficients, and we used the partial correlation test to assess for correlations while controlling for malaria severity. Differences in proportions between groups were assessed with Chi-square test. Multivariate linear regression was used to control for confounding variables that could affect urine BH_4_ and plasma phenylalanine concentrations. Bivariate logistic regression was used to analyze continuous variables for co-variation by a binomial variable (e.g., co-variation of urine BH_4_ concentration by the presence or absence of pyuria). For children with UM and CM, backward stepwise multivariate logistic regression was performed to determine whether phenylalanine or urine biopterin species were independently associated with disease severity. Bivariate logistic regression was performed for available biologically plausible variables known to be associated with severe malaria: hemoglobin, PfHRP-2, and respiratory rate and bicarbonate, as indicators of metabolic acidosis [3,71,72]. Variables were included in the multivariate logistic regression model if p < 0.20 on bivariate analysis, and were retained in the multivariate model if the p-value generated in the multivariate analysis was <0.05. The multivariate model also controlled for age, sex, weight and duration of fasting. Plasma creatinine was not included in the multivariate model because creatinine results were only available for 14 UM and 34 CM patients with urine biopterin results. In a multivariate logistic regression sub-model of these 48 malaria patients we did include creatinine in order to assess the relationship between estimated renal function and odds of cerebral malaria. Continuous variables with non-parametric distributions were log-transformed to meet normality assumptions for use in the logistic regression models. Goodness-of-fit was assessed by Hosmer-Lemeshow test. A two-sided p-value of < 0.05 was employed as the cut-off for statistical significance throughout the manuscript unless otherwise indicated (e.g., Bonferoni adjustments when making comparisons across multiple groups).

## Supporting Information

S1 Fig Scatterplot correlation of plasma Angiopoeitin-2 and urine dihydrobiopterin. Plasma Angiopoeitin-2 (Ang-2) plotted against urine dihydrobiopterin (BH_2_). Squares represent healthy controls (HC, n = 9), triangles represent uncomplicated malaria (UM, n = 5), circles represent cerebral malaria (CM, n = 13), and stars represent non-malaria central nervous system conditions (NMC, n = 9).
(TIF)

S2 Fig Representative chromatograms of biopterin and neopterin analytes. **A**: Electrochemical detection of of tetrahydrobiopterin (BH_4_) and dihydroneopterin (NH_2_) in urine. **B**: Electrochemical detection of BH_4_ and NH_2_ in standards. **C**: Fluorescence detection of neopterin (N_0_), dihydroneopterin (NH_2_), biopterin (B_0_), and dihydrobiopterin (BH_2_) in urine. **D**: Fluorescence detection of N_0_, NH_2_, B_0_, and BH_2_ in standards.
(TIF)
