# Supplementary figures and images for: Impaired Systemic Tetrahydrobiopterin Bioavailability and Increased Oxidized Biopterins in Pediatric Falciparum Malaria: Association with Disease Severity
Source: PLoS Pathog. 2015 Mar 12;11(3):e1004655. doi: 10.1371/journal.ppat.1004655 (PMC4357384; doi:10.1371/journal.ppat.1004655)

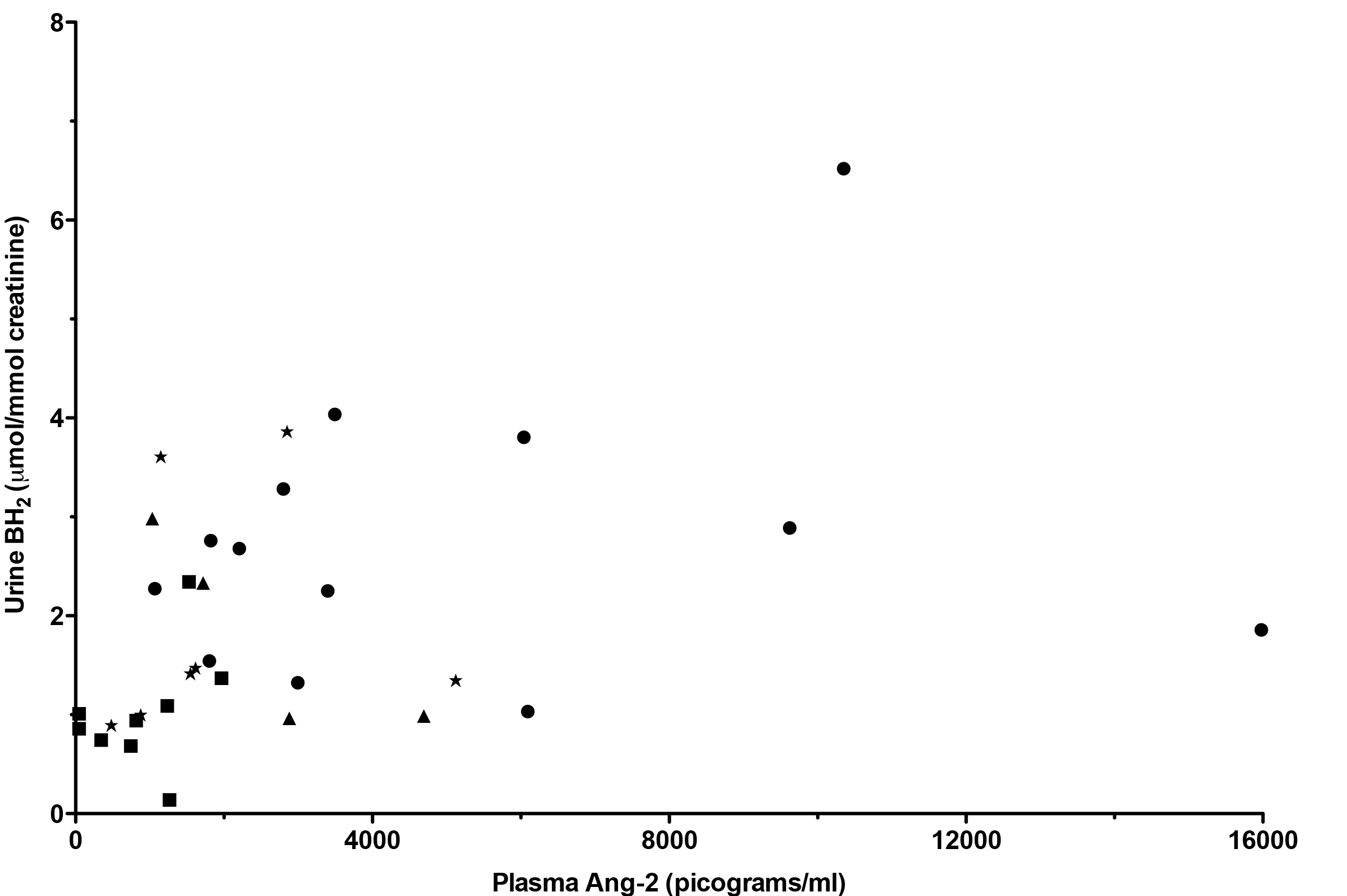

Supplement: S1 Fig — Plasma Angiopoeitin-2 (Ang-2) plotted against urine dihydrobiopterin (BH2). Squares represent healthy controls (HC, n = 9), triangles represent uncomplicated malaria (UM, n = 5), circles represent cerebral malaria (CM, n = 13), and stars represent non-malaria central nervous system conditions (NMC, n = 9). (TIF) [file ppat.1004655.s001.tif]

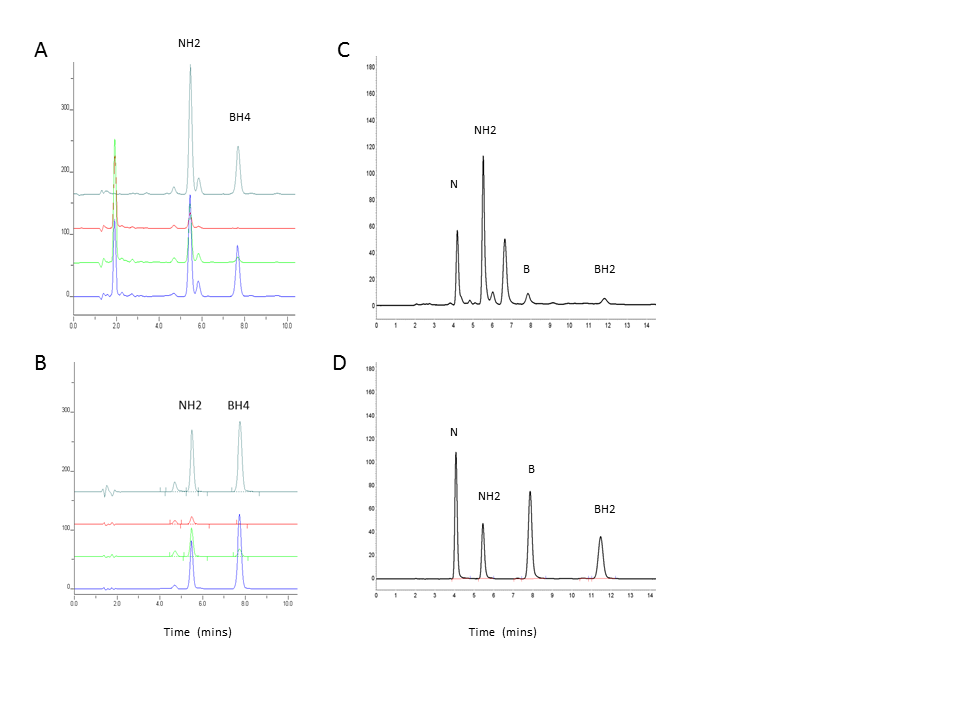

Supplement: S2 Fig — A: Electrochemical detection of of tetrahydrobiopterin (BH4) and dihydroneopterin (NH2) in urine. B: Electrochemical detection of BH4 and NH2 in standards. C: Fluorescence detection of neopterin (N0), dihydroneopterin (NH2), biopterin (B0), and dihydrobiopterin (BH2) in urine. D: Fluorescence detection of N0, NH2, B0, and BH2 in standards. (TIF) [file ppat.1004655.s002.TIF]
